# High Polyphenol Extra Virgin Olive Oil and Metabolically Unhealthy Obesity: A Scoping Review of Preclinical Data and Clinical Trials

**DOI:** 10.3390/clinpract15030054

**Published:** 2025-03-07

**Authors:** Konstantina Liva, Athanasios A. Panagiotopoulos, Alexandra Foscolou, Charalampia Amerikanou, Alkistis Vitali, Stavros Zioulis, Konstantina Argyri, Georgios I. Panoutsopoulos, Andriana C. Kaliora, Aristea Gioxari

**Affiliations:** 1 Department of Nutritional Science and Dietetics, School of Health Sciences, University of the Peloponnese, Antikalamos, 24100 Kalamata, Greece; k.liva@go.uop.gr (K.L.); ath.panagiotopoulos@go.uop.gr (A.A.P.); alexandra.foscolou@go.uop.gr (A.F.); kargyri@uop.gr (K.A.); gpanouts@uop.gr (G.I.P.); 2 Department of Dietetics and Nutritional Science, School of Health Science and Education, Harokopio University, 70 El. Venizelou Ave., 17676 Athens, Greece; camer@hua.gr (C.A.); akaliora@hua.gr (A.C.K.)

**Keywords:** extra virgin olive oil, polyphenols, obesity, metabolic syndrome, hydroxytyrosol, tyrosol, oxidative stress, inflammation

## Abstract

**Background/Objectives**: During the last decade, there has been an increased interest in phenolic compound-rich natural products as natural therapies for regulating the molecular pathways behind central obesity and associated metabolic disorders. The present scoping review presents the outcomes of clinical and preclinical studies examining the anti-obesity effects of high phenolic extra virgin olive oil (HP-EVOO) and its possible underlying molecular mechanisms. **Methods:** Studies published between 2014 and 2024 were searched via MEDLINE, Scopus, Cochrane, the Web of Science, Semantic Scholar, Google Scholar, Science.gov, and Clinicaltrials.gov databases. A combination of keywords and Boolean logic was used to search throughout the last decade in all databases, including “hyperglycemia” or “hypertension” or “metabolic syndrome” or “dyslipidemia” or “hyperlipidemia” or “hypoglycemia” or “obesity” or “macrovascular diabetic complications” or “microvascular diabetic complications” or “cardiovascular disease” or “overweight” or “insulin sensitivity” or “insulin resistance” and “extra virgin olive oil” or “high phenolic olive oil” and “human” or “animal model”. **Results**: The 10-year literature survey identified 21 studies in both animal models and humans, indicating that HP-EVOO improves inflammation, glycemic control, oxidative stress and endothelial function, potentially protecting against metabolic syndrome, hypertension and type 2 diabetes, even compared to EVOO. Moreover, HP-EVOO’s antiplatelet effect and improvement in HDL functionality reduce cardiovascular risk. **Conclusions**: The evidence presented in this study demonstrates that HP-EVOO represents an effective preventive and therapeutic dietary approach to cardiometabolic diseases.

## 1. Introduction

Obesity is a multifactorial disorder characterized by excessive accumulation of body fat as a result of energy intake exceeding energy expenditure, which threatens human health [1]. It is well documented that non-desirable weight gain and obesity contribute to the global rise in non-communicable diseases (NCDs) such as cardiovascular diseases (CVDs), diabetes, hypertension, chronic respiratory diseases, cancer, and neurological disorders (e.g., dementia, Alzheimer’s’, Parkinson’s disease) [2,3]. The latest World Health Organization report shows that the global prevalence of obesity has at least doubled over the past 30 years, with over 2.5 billion adults living with overweight or obesity by 2022 [4,5,6].

A plethora of genetic, epigenetic, physiological, and environmental factors affect food intake, which in turn may promote overweight and obesity [7]. Inflammation is considered as the main underlying mechanism for the development of metabolic disorders related to obesity, including dyslipidemia, insulin resistance, diabetes, and hypertension, as well as metabolic syndrome [7]. Metabolic syndrome (MetS) is defined as the concurrence of at least three obesity-associated cardiovascular risk factors: (a) abdominal obesity; (b) dysglycemia (impaired glucose tolerance and/or fasting glucose); (c) dyslipidemia (hypertriglyceridemia and/or low high-density lipoprotein (HDL)); and (d) hypertension [3,8]. Patients with MetS are at high risk of developing type 2 diabetes and cardiovascular diseases [7,9]. In fact, there is evidence that fat accumulation in the adipose tissue triggers the production of pro-inflammatory mediators, e.g., tumor-necrosis factor-alpha (TNF-α) and interleukin-6 (IL-6) [7]. At the same time, adiponectin secretion in the adipose tissue decreases leading to dysregulation of energy, glucose, and lipid metabolism [7]. On the other hand, secretion of leptin (another adipokine) increases in people living with obesity, which has been suggested to dysregulate systemic metabolism and appetite. There is evidence that leptin influences immune cell function as well [10,11]. A reduction in the adiponectin-to-leptin ratio is associated with insulin resistance and the development of type 2 diabetes [10]. It is noteworthy that about one-third of US adults and nearly 25% of the globally grown population suffer from MetS [12,13] and thus, urgent attention is needed.

Manipulation of dietary intake is a common way to manage overweight and obesity. Previous randomized-controlled trials (RCTs) have shown that high adherence to plant-based diets, such as the Mediterranean dietary pattern, is associated with improved metabolic health [14]. High adherence to the Mediterranean diet can lower body weight (BW) and body mass index (BMI) compared to other dietary patterns, resulting in decreased total body fat, as well as improved lipidemic profile, insulin sensitivity and arterial blood pressure [15,16,17]. The favorable metabolic effects of the Mediterranean diet in overweight/obesity are accompanied by lower inflammatory and oxidative stress responses [17,18,19], as well as by regulation in adipokine production [10,19]. These observations are probably attributed to the high content of bioactive substances such as polyphenols in the Mediterranean diet [20].

Polyphenols are secondary metabolites of plants, forming a large number of different substances with aromatic ring(s) and hydroxyl groups [20]. Polyphenols, as intrinsic antioxidants, have been claimed to be nutraceuticals (bioactive ingredients, naturally occurring and extractable from different food sources). It is well documented that polyphenols prevent overgeneration of oxidant species in normal cells and counteract oxidative stress in chronic diseases [21]. Since antiquity, olive oil has had a central role in the diet of Mediterranean populations [22]. The beneficial effects of olive oil, especially extra virgin olive oil (EVOO), have been well described in numerous studies, highlighting its contribution to the prevention of cardiovascular diseases, type 2 diabetes, and other chronic conditions [22]. EVOO is a major source of monounsaturated fatty acids (MUFAs) with a high cardioprotective impact [23]. The Primary Prevention of Cardiovascular Disease with Mediterranean Diet (PREDIMED) study, with a total of 7447 enrolled volunteers, highlighted that the addition of EVOO to the Mediterranean diet reduces the risk of major CV events [24]. Nowadays, EVOO is renowned for its rich polyphenol content, contributing to the unique flavor and aroma of the product. Overall, there are 25 polyphenols in EVOO, including hydroxytyrosol, oleuropein, oleocanthal and oleacein [25]. The majority of in vitro and in vivo studies have shown that the cardioprotective effect of EVOO is highly attributed to its polyphenol content [23,26].

For example, hydroxytyrosol (3,4-dihydroxyphenylethanol) is among the most potent antioxidants in EVOO, and it has the capacity to prevent in vitro macrophage oxidation of LDL [27]. This observation is reinforced by the results of in vivo studies where hydroxytyrosol intake was associated with an improvement in blood lipid profile, plaque formation, and inflammatory status [27]. This phenolic molecule is also thought to have anti-atherogenic effects by decreasing the expression of intercellular adhesion molecule 1 (ICAM-1) and vascular cell adhesion protein 1 (VCAM-1) in isolated aorta from apolipoprotein E knockout mice [27]. Oleuropein, another polyphenol contained in significant amounts in EVOO, has been shown to reduce blood levels of fasting glucose, low-density lipoprotein (LDL), and triglycerides (TGs) and to improve endothelial function in vivo [27,28]. It has been suggested that oleuropein regulates glucose metabolism, as indicated by favorable changes in glucose transport and intracellular metabolism, increased insulin sensitivity, and more efficient glucose-stimulated insulin secretion by the pancreatic cells [27]. EVOO also contains 3,4 dihydroxyphenylglycol (DHPG), posited to enhance antioxidant and anti-inflammatory effects in a diabetic rat model [29]. Furthermore, in vivo and in vitro anti-inflammatory, cardioprotective, chemoprotective, and neuroprotective properties have been attributed to EVOO phenolic compounds including tyrosol, oleocanthal, oleasin, and ligstroside aglycone [30].

In May 2012, the European Food Safety Agency (EFSA) authorized the claim that “olive oil polyphenols contribute to the protection of blood lipids from oxidative stress”. This claim was validated for olive oils that contain at least 5 mg of hydroxytyrosol and its derivatives per 20 g of olive oil [31]. Thus, by definition, high phenolic EVOO (HP-EVOO) contains at least 250 mg of polyphenols per kilogram of oil [31].

During the last decade, research interest on the health-promoting properties of HP-EVOO has been increasing [32]. Till now, a small but substantial number of studies investigating the effects of HP-EVOO on metabolically unhealthy obesity have been completed, yet the results are still unclear [21,33]. Therefore, in this scoping review, we aim to present the outcomes of RCTs and animal studies of the last decade, which tried to identify the role of HP-EVOO in obesity and obesity-related metabolic disorders, and shed light on the underlying mechanism of HP-EVOO in metabolic homeostasis.

## 2. Materials and Methods

An extensive literature search was conducted, using the following databases: MEDLINE, Scopus, Cochrane, Web of Science databases, Semantic Scholar, Google Scholar, Science.gov, and Clinicaltrials.gov. This review followed the PRISMA 2020 guidelines for systematic reviews and scoping reviews. In this review, we applied the following inclusion criteria:

- Original RCTs and animal studies,
- Studies investigating the effects of HP-EVOO in which (a) the concentration of total phenolic compounds is above 250 ppm or (b) the concentration of specific phenolic compounds is enhanced,
- Articles written in English,
- Articles published between 2014 and 2024.
- Studies were excluded if they met any of the following criteria:
- Reviews, meta-analyses, in vitro experimental studies, unpublished or ongoing in vivo studies, and clinical trials,
- Full-text articles that were not retrieved or not written in English,
- Studies that examined diseases unrelated to obesity and metabolic abnormalities, such as autoimmune disorders, cancer, chronic respiratory diseases, or neurological diseases or studies conducted on hospitalized patients,
- Studies involving other natural products,
- Studies conducted on children or pregnant women.

The literature search employed a combination of keywords and Boolean operators (such as AND, OR, and NOT) across all databases, including “hyperglycemia” or “hypertension” or “metabolic syndrome” or “dyslipidemia” or “hyperlipidemia” or “hypoglycemia” or “obesity” or “macrovascular diabetic complications” or “microvascular diabetic complications” or “cardiovascular disease” or “overweight” or “insulin sensitivity” or “insulin resistance” and “extra virgin olive oil” or “high phenolic olive oil” and “human” or “animal model”. References from the extracted articles were also used.

To ensure the rigor, trustworthiness and transparency of the findings, the PICO framework (Population, Intervention, Comparison, Outcome) was utilized to structure the selection process of human studies. The relevance of the studies was assessed by a hierarchical approach, beginning with the title, abstract, and full-text review.

**Figure 1:**
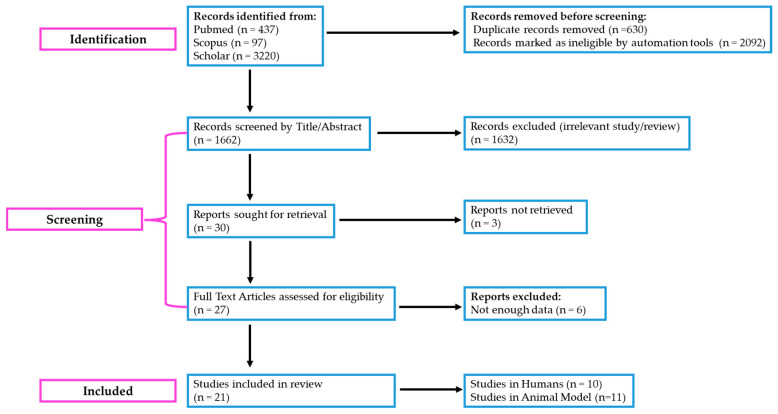
“PRISMA” 2020 flow diagram illustrating the literature selection process for the scoping review on HP-EVOO and its effects on obesity and metabolic abnormalities. The diagram follows the PRISMA Extension for Scoping Reviews (PRISMA-ScR) guidelines to ensure transparency and rigor in reporting. Adapted from the PRISMA 2020 Flow Diagram (https://www.prisma-statement.org/prisma-2020-flow-diagram) (assessed on 30 January 2025).

The information and outcomes of each study were extracted and reported in a standardized format, including the following: (i) citation, author, year of publication, and study purpose, (ii) inclusion and exclusion criteria, (iii) type of intervention and the amount and type of phenolics used, (iv) sample size, and (v) baseline and post-intervention metabolic variables [34,35]. A flow diagram illustrating the study selection process is presented in Figure 1.

## 3. Results of Preclinical and Clinical Studies

**Figure 2:**
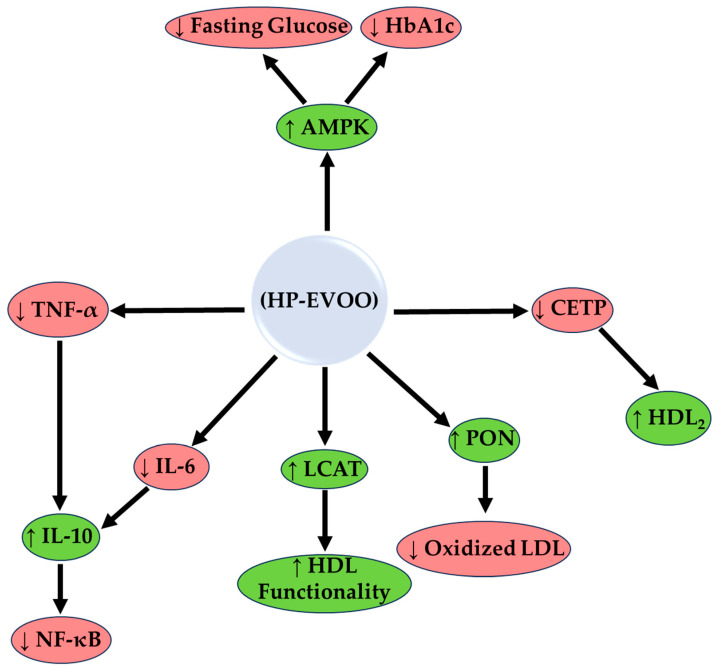
The enzymological mechanism of action of high phenolic extra virgin olive oil (HP-EVOO). The mechanism is based on preclinical and clinical data. HP-EVOO enhances antioxidant activity through the activation of Paraoxonase (PON) and Lecithin-Cholesterol Acyltransferase (LCAT), reducing oxidized LDL and improving HDL functionality. In addition, HP-EVOO polyphenols inhibit the inflammatory activity of NF-κB, reducing inflammatory markers (IL-6, TNF-α) and increasing anti-inflammatory ones such as IL-10. In the lipid pathway, CETP inhibition increases HDL_2_, while in the glycemic pathway, activation of AMPK reduces fasting glucose and HbA1c; ↑, increase: green shapes; ↓, decrease: red shapes.

The 10-year literature survey resulted in 21 publications that investigated the effect of HP-EVOO in humans (Table 1) and animals (Supplementary Table S1). In summary, the main mechanism of biological action of HP-EVOO based on preclinical and clinical data is presented in Figure 2.

### 3.1. Preclinical Studies

#### 3.1.1. Effects on Body Weight and Body Composition

Consumption of HP-EVOO has beneficial effects on body weight management and body composition in animals. In overweight mice fed on a Western-like diet, substitution of saturated fat for HP-EVOO was associated with a decrease in overall weight gain by 11.5% [36]. HP-EVOO reduced body weight gain compared to high-fat diet (HFD) and lard-fed groups [36,37,38,39,40] but had no significant effect compared to mice consuming EVOO [38]. Protection against adipose tissue hypertrophy is another property of HP-EVOO intake [36]. In LDL-receptor knockout Leiden mouse models (*LDLr−/−*) with non-alcoholic fatty liver disease (NAFLD) and obesity, HP-EVOO intake significantly reduced adipose tissue hypertrophy [36,37,38,39,41,42]. Fat cell dimensions were smaller in the HP-EVOO groups than in the lard-fed groups [39]. This observation was accompanied by a lower inflammatory response in the adipose tissue, as indicated by lower levels of macrophage influx [39]. Consumption of HP-EVOO reduced the severity of atherosclerotic lesions in HFD animals [17,28,39]. In animal groups consuming HP-EVOO, collagen concentration and aortic valve stenosis decreased, suggesting that HP-EVOO contributes to lower cardiometabolic risk under conditions of high fat intake [39]. In contrast to mice on HFD, atherosclerotic lesions were not lessened in mice consuming extra virgin olive oil with HFD. However, the aortic production of nitrites and nitrates was decreased when the phenolic content of extra virgin olive oil was increased. In addition, HP-EVOO lowered the blood concentration of TG and free fatty acids, which in turn reduced fat deposition in the liver compared to EVOO [40,43]. Dietary supplementation with HP-EVOO lessened the negative effects of HFD in mice by reducing oxidative stress, maintaining ω-3 long-chain polyunsaturated fatty acids (LCPUFAs) synthesis, and preventing tissue depletion of these fatty acids. These findings indicate that HP-EVOO consumption supports a lean body profile through mechanisms that involve the reduction in inflammation, lipid synthesis, and deposition [40,43].

#### 3.1.2. Glycemic Regulation and Insulin Sensitivity

HP-EVOO consumption is related to beneficial effects on glycemic regulation [37,38,44,45] and improved insulin sensitivity, protecting pancreatic β-cells against dysfunction and apoptosis in rodents fed on an HFD [36,37]. Compared to standard diets or EVOO, consumption of HP-EVOO demonstrated a greater impact on reducing fasting glucose and insulin levels, contributing to improved glucose homeostasis [38]. Rats with alloxan monohydrate-induced diabetes showed high hypoglycemic activity after ingestion of HP-EVOO [41]. In the study by Jurado-Ruiz and co-authors, HP-EVOO and EVOO diets lowered fasting glucose and insulin levels, maintaining, therefore, glucose homeostasis in mice fed on an HFD [38]. Nevertheless, no differences were observed between EVOO and HP-EVOO groups. Both diets improved insulin secretion in response to the intraperitoneal glucose tolerance test (IPGTT), suggesting a better β-cell response to high glucose levels compared to lard [38]. The authors suggested that MUFAs play a key role in glucoregulation by modifying (a) membrane free fatty acid composition in pancreatic islets, (b) membrane fluidity, (c) the interaction with cell-surface or intracellular receptors, and (d) endoplasmic reticulum stress.

On the other hand, Lama and co-authors attributed the beneficial effects of HP-EVOO on glycemic control to the high polyphenol content [45]. In fact, HP-EVOO improved insulin sensitivity in obese rats on an HFD, as manifested by a decrease in the insulin resistance index and the homeostatic model assessment for insulin resistance (HOMA-IR) and an increase in activity of the insulin degrading enzyme (IDE) in the liver. These observations probably contributed to the restoration of insulin levels in HP-EVOO animals compared to those consuming an HFD or olive oil without polyphenols [45]. Within this context, studies observed activation of the serine-threonine protein kinase 1 (AKT) pathway and increased expression of glucose transporter 2 (GLUT2; low-affinity for glucose transporter) in the liver, suggesting that HP-EVOO is more efficient at promoting glucose transport and enhancing metabolic response compared to HFD or olive oil without polyphenols [45].

A diet with HP-EVOO or EVOO seems to protect the pancreatic β-cells by diminishing apoptosis and increasing the number and functionality compared to lard-rich diets [38]. Moreover, compared to HFD-fed mice, HP-EVOO and EVOO groups showed a better ability of β-cells to secrete insulin in response to glucose [38]. Though most of the key roles of olive oil had been largely assigned to MUFAs, the presence of polyphenols enhanced metabolic homeostasis in HFD-fed animals [37,38,45]. This would suggest that HP-EVOO could be used as a means to improve glycemic regulation by increasing insulin sensitivity and functionality of β-cells.

#### 3.1.3. Blood Lipid Profile and Cholesterol Regulation

HP-EVOO contains a high amount of MUFAs and polyphenols, which have a beneficial effect on the blood lipid profile and cholesterol homeostasis, thereby reducing metabolic and CVD risks [40]. Consumption of HP-EVOO by NAFLD mice has been shown to reduce total cholesterol and LDL levels, while HDL concentration was significantly increased compared to lard- or olive oil-fed mice [37,39,40,41,42,45]. In experiments using *LDLr−/−* Leiden mice fed with an HFD, the addition of HP-EVOO decreased the severity of atherosclerotic plaques and increased levels of adiponectin [39]. A rise in adiponectin concentration is associated with improved vascular health and less inflammation within the endothelium [39]. Compared to other groups, HP-EVOO had a more pronounced effect on lipid metabolism.

In high-cholesterol diet models, HP-EVOO outperformed phenolic-deprived EVOO in reducing inflammation and oxidative stress [46]. HP-EVOO also showed a more significant reduction in malonhydelhide (MDA) levels than EVOO deprived of its polyphenols, highlighting its superior antioxidant capacity [46]. HP-EVOO also showed a greater reduction in blood TGs than HFD and olive oil without polyphenols [40,43]. Compared to HFD-fed mice, HP-EVOO resulted in more substantial decreases in fatty infiltration and hyperlipidemia [40,43]. These results reinforce the superior role of HP-EVOO in cardiovascular protection through improved lipid metabolism. Rats being on a high-cholesterol diet benefited from the anti-inflammatory and antioxidant effects of EVOO polyphenols [46].

#### 3.1.4. Antioxidant Action and Regulation of Oxidative Stress

Compared to HFD and standard EVOO intake, HP-EVOO exhibited greater antioxidant effects by significantly reducing oxidative stress markers such as glutathione disulfide (GSSG) and MDA [40,43,46]. HP-EVOO completely suppressed GSSG levels while maintaining GSH concentration, and this was more evident when compared to EVOO [40,43]. Additionally, HP-EVOO reduced F2-isoprostane levels more effectively than other dietary oils, highlighting its potent antioxidative action [43].

In models of high-fat diets, HP-EVOO showed greater improvement in mitochondrial function compared to EVOO and other oils, reducing ROS production and increasing aconitase activity, which enhances mitochondrial efficiency [45]. Compared to high-cholesterol diets, HP-EVOO demonstrated superior antioxidant protection by limiting lipid peroxidation and elevating GSH levels [46]. These findings highlight the protective effects of HP-EVOO over EVOO (and other dietary oils) against oxidative damage.

HP-EVOO has been shown to promote fatty acid balance by (a) increasing the production of omega-3 polyunsaturated fatty acids (PUFAs) such as docosahexaenoic acid (DHA), (b) limiting DHA loss in the brain, and (c) protecting against lipid oxidation in vital tissues such as the liver, adipose tissue, and heart [43]. In rats fed with a high-cholesterol diet, EVOO polyphenols reduced lipid peroxidation (MDA levels) and enhanced natural antioxidant parameters (GSH levels), providing heart protection compared to high-cholesterol-fed rats [46]. These data show that EVOO polyphenols are key components of an antioxidant diet, with the potential to protect against chronic inflammatory and metabolic disorders related to obesity [46].

#### 3.1.5. Liver Health and Fatty Infiltration

Consumption of HP-EVOO has been shown to offer protection to the liver by reducing fatty infiltration, improving fat metabolism, and reducing inflammation and damage associated with NAFLD and non-alcoholic steatohepatitis (NASH) [40,43]. Compared to lard and standard EVOO, consumption of HP-EVOO demonstrated a protective effect on the liver by significantly reducing fatty infiltration and fibrosis [37,39,40,43,45]. HP-EVOO-fed mice had a mean nonalcoholic steatohepatitis (NAS) score of 2, compared to 4.67 in lard-fed mice [40]. HP-EVOO was more effective in improving hepatic fatty acid composition than EVOO, as indicated by reduced SFAs and TGs and increased MUFAs [40,43]. HP-EVOO also modulated liver gene expression more effectively than EVOO by increasing PPAR-α and Cd36 and down-regulating inflammatory and fibrosis-related genes like COX-2 and TNF-α [40,42].

Compared to a control diet or olive oil diet of low polyphenol content, HP-EVOO supplementation enhanced the expression of genes responsible for lipid metabolism such as PPAR-α and cluster of differentiation 36 (Cd36), which increased lipolysis and fatty acid oxidation [43]. Additionally, there was a down-regulation in the expression of genes of inflammatory and fibrosis markers, i.e., cyclooxygenase-2 (COX-2), inducible nitric oxide synthase (iNOS), and tumor necrosis factor alpha (TNF-α); this suggests that HP-EVOO exerts hepatoprotection through the regulation of gene expression (Figure 2) [40,42]. In the models of NAFLD/NASH, HP-EVOO alleviated liver inflammation by reducing the inflammatory macrophage infiltration and expression of cytokines like interleukin-6 (IL-6) compared to an HFD with lard or olive oil [40].

These findings suggest that HP-EVOO supplementation restricts the inflammatory response of the liver and prevents the development of fibrosis [43]. HP-EVOO may be one of the most relevant dietary elements in the prevention and management of metabolic dysfunction–associated steatotic liver disease [40,43].

#### 3.1.6. Inflammation and Immune Regulation

EVOO, and especially HP-EVOO, exerts a considerable anti-inflammatory effect, modulating the regulation of inflammatory response and immune balance [40,42,46]. Compared to HFD and EVOO, consumption of HP-EVOO demonstrated greater anti-inflammatory effects, as indicated by significantly decreased TNF-α, IL-6, and COX-2 [40,42,46]. HP-EVOO increased IL-10 and adiponectin levels more effectively compared to EVOO, highlighting its role in immune regulation [39]. In the adipose tissue, HP-EVOO limited macrophage infiltration compared to standard EVOO [40]. Moreover, HP-EVOO reduced the expression of the pro-inflammatory cytokines TNF-α and IL-6, compared to HFD and EVOO [40,42,46]. At the same time, HP-EVOO intake decreased the expression of enzymes COX-2 and iNOS, which are associated with the production of prostaglandins and nitric oxide [42]. Compared to HFD or olive oil without polyphenols, HP-EVOO intake increased the secretion of anti-inflammatory IL-10 [45] (Figure 2) and adiponectin [39], which both mitigate inflammation. These actions have been observed in various tissues:

(a) In adipose tissue, HP-EVOO compared to lard or EVOO consumption reduced the infiltration of inflammatory macrophages and limited the formation of crown-like structures (CLSs), which are associated with chronic inflammatory processes [40];
(b) In the liver, HP-EVOO compared to lard or EVOO reduced the presence of inflammatory cytokines, such as interleukin 1β (IL-1β), and reduced the development of fibrosis [40,43];
(c) In the large intestine, HP-EVOO, compared with EVOO, showed a reduction in pro-inflammatory markers and an increase in antioxidant defenses, along with reduced histopathological tissue damage [42].

These findings suggest that the consumption of HP-EVOO, compared with EVOO, provides multi-level protection and enhanced overall immune balance [42].

#### 3.1.7. Protection of the Heart and Reduction in Cardiovascular Risk

Compared to HFD, the observed cardioprotection of HP-EVOO is mainly attributed to the reduced inflammatory and oxidative stress response, as well as the improved overall heart function [46,47]. Regular consumption of EVOO, and particularly HP-EVOO, seems to decrease inflammatory markers implicated in the atherosclerosis process such as TNF-α [48], along with a reduction in ROS, which causes oxidative damage to the heart cells [46]. Compared to standard EVOO and HFD, consumption of HP-EVOO showed stronger cardioprotective effects by significantly reducing TNF-α and MDA in the heart tissue [46]. In addition, HP-EVOO was more effective in increasing IL-10 levels [45]. Compared to high-cholesterol diets, HP-EVOO reduced oxidative stress more effectively, showing greater protection against atherosclerosis [46]. Moreover, HP-EVOO activated the AMPK pathway and increased PPAR-α expression more significantly than standard EVOO, enhancing fatty acid oxidation and reducing heart lipid accumulation [45]. In another study, EVOO’s oleic compounds and phenolic components were found to improve endothelial function in cholesterol-fed rats, but polyphenol enrichment of olive oil did not affect further aortic tissues [49].

Nonetheless, the above findings highlight the strong anti-inflammatory and antioxidant activities of HP-EVOO, promoting cardiometabolic health [45,46].

### 3.2. Clinical Studies

#### 3.2.1. Effects on Body Composition Metrics

Olive oil, particularly HP-EVOO, has been shown to favorably affect both body weight and fat distribution [50,51,52]. The phenolic compounds found in olive oil are believed to modulate metabolic pathways that control lipid metabolism, fat oxidation, and appetite regulation. Additionally, olive oil polyphenols seem to reduce chronic low-grade inflammation, which is linked to obesity and insulin resistance [50,51,52]. In research conducted by Santangelo et al. (2016), a total of 11 overweight patients diagnosed with type 2 diabetes participated in the study [52]. Participants consumed 25 mL of HP-EVOO daily (577 mg/kg of phenolic compounds) for four weeks [52]. The results showed a significant weight reduction by 0.97 kg (*p* = 0.012) compared to participants consuming refined olive oil, indicating the potential of olive oil to contribute to weight loss without calorie restriction [52]. In the two-month intervention of Patti et al. (2020) involving 23 patients with MetS and moderate hepatic steatosis, daily consumption of 32 g of HP-EVOO resulted in 2 kg weight loss (*p* = 0.035), BMI reduction by 1 unit (*p* = 0.031), decrease in waist circumference by 3 cm (*p* = 0.037), and improvement in visceral-to-subcutaneous fat ratio (*p* = 0.020), compared to baseline [51]. These findings suggest that HP-EVOO can specifically target visceral fat, a key factor in metabolic disease risk [51]. In the 1-month randomized crossover study of Ruiz-García et al. (2023) involving 91 participants with obesity, HP-EVOO consumption led to significant weight loss (approximately 1 kg, *p* < 0.05) and BMI reduction compared to olive oil, even without altering dietary or physical activity habits [50].

Therefore, regular consumption of HP-EVOO, can modestly but significantly contribute to weight management and improved body composition, particularly in individuals with metabolic disorders.

#### 3.2.2. Effects on Markers of Glycemic Regulation

HP-EVOO has demonstrated benefits in stabilizing fasting blood glucose levels, improving insulin sensitivity, and reducing glycemic spikes after meals. According to Santangelo et al. (2016), in patients with type 2 diabetes, daily consumption of HP-EVOO significantly reduced fasting glucose levels (*p* = 0.023) and HbA1c (glycated hemoglobin) (*p* = 0.039), indicating long-term improvements in blood glucose control compared to refined olive oil [52]. In the study of Ruiz-García et al. (2023), HP-EVOO intake significantly lowered fasting glucose levels (*p* < 0.05) of participants with obesity and prediabetes compared to olive oil [50]. However, there were no significant changes in HbA1c or insulin resistance markers during the short study period [50].

According to the forementioned outcomes, it seems that HP-EVOO rich in phenolic compounds like oleuropein and hydroxytyrosol improves insulin sensitivity by modulating insulin receptor signaling [50,52,53]. Moreover, HP-EVOO reduces postprandial glycemic response by slowing gastric emptying and enhancing cellular glucose uptake [50,52,53].

#### 3.2.3. Effects on Blood Lipid Biomarkers

HP-EVOO is known for its beneficial effects on lipid metabolism, reducing cholesterol levels and improving the functionality of HDL [53,54,55,56,57]. According to the study of Farràs et al. (2015) in hypercholesterolemic patients, daily consumption of 25 mL HP-EVOO led to a 5.74% increase in HDL and 34.45% increase in the HDL_2_ subclass compared to EVOO (*p* < 0.05). Additionally, patients receiving HP-EVOO showed a reduction in CETP activity (Cholesteryl Ester Transfer Protein), a key enzyme involved in cholesterol transfer, and enhancement in LCAT (Lecithin-Cholesterol Acyltransferase) and PON (Paraoxonase) enzyme activity, which indicate better HDL functionality compared to patients receiving EVOO [54]. In healthy adults, HP-EVOO resulted in increased HDL cholesterol levels (*p* = 0.041) compared to EVOO, while stronger effects were recorded in female participants (*p* = 0.005) [55]. Compared to refined olive oil, HP-EVOO reduced total cholesterol by 9.52 mg/dL (*p* = 0.007) and LDL by 5.10 mg/dL (*p* = 0.011) in participants at cardiovascular risk. Additionally, HP-EVOO led to a lower CRP (*p* = 0.01), a potent inflammatory marker, than the refined olive oil [56]. In the study of Katsa et al. (2024), oleocanthal-rich oils resulted in LDL reductions by 4–10% and slight decreases in HDL levels compared to butter, butter and ibuprofen, olive oil and EVOO groups [53]. Moreover, the study of Martín-Peláez et al. (2016) demonstrated that HP-EVOO consumption by hypercholesterolemic adults reduced oxidized LDL (*p* = 0.005), indicating improved lipid peroxidation compared to olive oil [57]. The mechanism behind these effects is attributed to the phenolic compounds of HP-EVOO that improve lipid metabolism and reduce oxidative damage, thus preventing plaque formation and increasing HDL functionality [53,54,55,56,57].

#### 3.2.4. Effects on Antioxidant Markers and Lipid Peroxidation

Oxidative stress is the cause of many chronic diseases. HP-EVOO’s antioxidant properties are crucial in reducing cellular damage caused by free radicals. In the study of Farràs et al. (2015), HP-EVOO significantly improved antioxidant activity within HDL, compared to olive oil [54]. Compared to EVOO or olive oil consumption by healthy adults, HP-EVOO increased levels of urine hydroxytyrosol, suggesting enhanced antioxidant activity [58]. According to the forementioned studies, phenolic compounds like hydroxytyrosol and oleuropein neutralize free radicals and improve antioxidant enzyme activity (e.g., superoxide dismutase, glutathione peroxidase) [54,58]. In conclusion, HP-EVOO, compared to EVOO or olive oil, reduces oxidative stress and prevents cellular damage, protecting from chronic disease progression.

#### 3.2.5. Effects on Liver Enzymes, Hepatic Steatosis, and Liver Function Markers

In clinical trials, consumption of HP-EVOO has shown beneficial effects on improving liver health, that is a reduction in fat accumulation, inflammation, and oxidative damage compared to baseline or refined olive oil [51,52]. In the two-month intervention of Patti et al. (2020) involving 23 patients with MetS and moderate hepatic steatosis, daily consumption of 32 g of HP-EVOO resulted in lower liver fat content (*p* = 0.043), improved liver enzyme levels (ALT and AST, *p* < 0.05) and better liver stiffness score (*p* = 0.037) compared to baseline [51]. In the study of Santangelo et al. (2016), 11 overweight patients with type 2 diabetes consumed 25 mL/day of HP-EVOO (577 mg/kg phenolics) for 4 weeks. The results showed that blood concentrations of aspartate aminotransferase (AST) and alanine aminotransferase (ALT) reduced (*p* < 0.05) compared to the olive oil group [52]. These findings suggest that HP-EVOO can reduce hepatic fat accumulation and improve liver function compared to baseline or olive oil [51,52]. As depicted in Figure 2, HP-EVOO polyphenols reduce inflammatory cytokines linked to liver damage, which in turn protect hepatocytes from oxidative stress, enhance fatty acid oxidation and lower hepatic lipid accumulation [51,52].

#### 3.2.6. Effects on Inflammatory Biomarkers and Cytokines

Chronic inflammation is a major cause of metabolic diseases, and olive oil’s phenolic compounds have been shown to have potent anti-inflammatory effects by lowering CRP, TNF-α, and IL-6 levels, [50,52,53,56,58]. According to the study of Khandouzi et al. (2021) in participants with cardiovascular risk factors, daily consumption of HP-EVOO reduced CRP levels by 22% (*p* = 0.01), while IL-6 significantly dropped (*p* = 0.015) compared to refined olive oil [56]. In the one-month study of Ruiz-García et al. (2023), HP-EVOO intake by participants with obesity resulted in enhanced levels of anti-inflammatory markers and reduced INF-γ compared the olive oil group [50]. Participants with type 2 diabetes who consumed oleocanthal-enriched HP-EVOO experienced enhanced antiplatelet effects, lower inflammatory cytokine levels (TNF-α, IL-1β, and IL-6, *p* < 0.05) and enhanced anti-inflammatory IL-10 concentration (*p* = 0.032) compared to butter, olive oil and EVOO groups [53]. Similarly, Santangelo et al. (2016) showed that consumption of HP-EVOO (577 mg/kg phenolics) by overweight and diabetic patients for 4 weeks led to lower levels of the inflammatory adipokine visfatin (*p* = 0.0021), while apelin, an anti-inflammatory adipokine, showed an increasing trend (*p* = 0.063) compared to olive oil [52].

#### 3.2.7. Effects on Blood Pressure, Endothelial Function, and Cardiovascular Risk Markers

Olive oil consumption is strongly linked to improved cardiovascular health with beneficial effects on blood pressure and arterial function [50,54,56,57,59]. In the study of Martín-Peláez, HP-EVOO decreased systolic blood pressure compared to the pre-intervention (baseline) values and the olive oil group, too [58]. Similarly, in the randomized, double-blind, crossover study of Sarapis et al. (2020), HP-EVOO (360 mg phenolics/kg) consumption by healthy adults for 3 weeks reduced peripheral systolic blood pressure by 2.5 mmHg (95% CI: −4.7 to −0.3, *p* < 0.05) and central systolic blood pressure by 2.7 mmHg (95% CI: −4.7 to −0.6, *p* < 0.05) compared to olive oil [59]. All these outcomes suggest that HP-EVOO polyphenols enhance nitric oxide (NO) bioavailability, leading to vasodilation and reduced arterial stiffness [50,54,56,57,59].

**Table 1:** Clinical data on the effects of high polyphenol extra virgin olive oil.

a/a	Ref.	Year	Population	Dosage	Design	Effect				
**1**	[53]	2024	T2DM patients	5 isocaloric meals containing 120 g white bread combined with (i) 39 g butter, (ii) 39 g butter and 400 mg ibuprofen, (iii) 40 mL OO (phenolic content < 10 mg/Kg), (iv) 40 mL OO with 250 mg/Kg oleocanthal, and (v) 40 mL OO with 500 mg/Kg oleocanthal	Acute, randomized, single-blinded, postprandial, crossover study, 10 T2DM patients. Blood samples were collected before and at 30, 60, 90, 120, 180, and 240 min after the meals	Biomarker	HP-EVOO 500 vs. Butter	HP-EVOO 500 vs. butter + ibuprofen	HP-EVOO 500 vs. OO	HP-EVOO 500 vs. HP-EVOO 250
						GLU, insulin, and C-peptide levels postprandially	↔	↔	↔	↔
						TG	↓	↓	↓	↓
						LDL-C	↓	↓	↓	↓
						HDL-C	↓	↓	↓	↓
						UA	↔	↔	↔	↔
						Homocysteine	↔	↔	↔	↔
						Antiplatelet effect (platelet activity indices ADP and TRAP)	↑	↔	↑	↑
**2**	[50]	2023	Adults 40–65 years old with obesity (BMI 30–40 kg/m^2^) and prediabetes (HbA1c 5.7–6.4%).	HP-EVOO with 508.4 mg/kg total biophenols vs. OO with 76.83 mg/kg. Secoiridoids constituting 93% of total biophenols in HP-EVOO (472.91 vs. 66.11 mg/kg. Oleocanthal (69%) and Oleacein (21%), 428.31 mg/kg in total). No specific amount of olive oil intake was indicated	randomized, double-blind, crossover trial in 91 people (33 men and 58 women) aged 40–65 years with obesity (BMI 30–40 kg/m^2^) and prediabetes (HbA1c 5.7–6.4%). The intervention consisted of substituting for 1 month the oil used for food, both raw and cooked, by HP-EVOO or OO	Biomarker	HP-EVOO vs. OO			
						INF-γ	↓			
						Anti-inflammation markers (CXCL1, IL-12p40 and IL-1RA)	↑			
						Total antioxidant status	↑			
						Lipid and organic peroxides	↓			
						BW	↓			
						BMI	↓			
						Blood GLU	↓			
**3**	[59]	2020	Healthy adults	60 mL/day HP-EVOO (360 mg/kg polyphenols) or OO (86 mg/kg polyphenols)	3 weeks, double-blind, cross-over trial, randomized controlled trial, 50 participants BMI = 18.5–40 kg/m^2^ (44% overweight), 2-week washout period while participants crossed-over to the alternate treatment	Biomarker	HP-EVOO vs. OO			
						Energy intake	↑			
						BMI, WC	↔			
						DBP	↔			
						Arterial stiffness	↔			
						Peripheral and central SBP	↓			
**4**	[56]	2021	Patients with at least one major cardiovascular risk factor: hypertension, diabetes mellitus, dyslipidemia, or acute cardiac events	25 mL of HP-EVOO 500–700 mg/kg or OO (refined) 2–1 mg/kg as raw with meals	6 weeks, randomized, controlled, parallel-arm, clinical trial, 40 men and post-menopausal women	Biomarker	HP-EVOO vs. OO (refined)			
						Energy intake	↔			
						TC, LDL-C	↓			
						Small dense-LDL-C, HDL-C, T-CHOL/HDL-C ratio, LDL-C/HDL-C ratio, TG	↔			
						Blood LPS-stimulated, IL-10 production	↑			
						IL-6, CRP levels	↓			
**5**	[51]	2020	European descent following a mediterranean diet, age between 18 and 70 years, the diagnosis of MetS as defined by international consensus and the presence of hepatic steatosis	4 large spoons of HP-EVOO with high oleocanthal concetration, daily (which corresponded to 32 g of HP-EVOO) during main meals	23 subjects with MetS and hepatic steatosis (15 men and 8 women, age: 60 years) for 2 months	Biomarker	After vs. Before HP-EVOO			
						BW	↓			
						WC	↓			
						ALT	↓			
						Inflammatory cytokines	↓			
						Hepatic steatosis	↓			
**6**	[55]	2018	Healthy adults	30 mL of (1) OO (124 ppm of phenolic compounds and 86 ppm of triterpenes); (2) HP-EVOO (490 ppm of phenolic compounds and 86 ppm of triterpenes); and (3) a functional EVOO (487 ppm) and enriched with triterpenes (389 ppm)	3-week randomized, crossover, controlled, double-blind, intervention study, 58 participants	Biomarker	HP-EVOO vs. Baseline	HP-EVOO vs. EVOO	HP-EVOO vs. Functional EVOO	
						HDL-C	↑	↑	↑	
						LDL-C	↑	↓	↓	
						TG	↑	↔	↑	
						Plasma ET-1	↓	↓	↑	
**7**	[58]	2017	Healthy adults	25 mL/day of olive oils (1) high (366 mg/kg, HP-EVOO) and (2) low (2.7 mg/kg, OO)	3 weeks, preceded by 2-week washout periods, randomized, double-blind, crossover human trial with 18 healthy subjects (BMI (kg/m^2^) 24.3 ± 3.2), waist/hip ratio 0.88 ± 0.06)	Biomarker	HP-EVOO vs. OO			
						SBP	↓			
						T-CHOL, LDL-C	↓			
						Oxidized LDL-C	↓			
						ACE, IL8RA, NR1H2 gene expressions	↓			
**8**	[57]	2016	Hypercholesterolemic participants	25 mL/day of 2 raw virgin OO differing in polyphenol concentration and origin: (1) an OO containing 80 mg polyphenol/kg (OO); (2) a polyphenol-enriched EVOO containing 500 mg polyphenol/kg (HP-EVOO)	3 weeks, randomized, controlled, double blind cross-over human trial, preceded by two-week washout periods, 10 hypercholesterolemic participants	Biomarker	HP-EVOO vs. OO			
						BMI, BW	↔			
						Oxidized LDL-C	↓			
						DBP, SBP	↔			
**9**	[52]	2016	Overweight T2D patients	Olive oil (OO refined, polyphenols not detectable) vs. HP-EVOO (25 mL/day, 577 mg of phenolic compounds/kg)	11 overweight T2D patients not in treatment with insulin were invited to follow their habitual diet for a total of 8 weeks. During the first 4 weeks (washout period), they were asked to consume refined olive oil and then to replace OO with HP-EVOO	Biomarker	HP-EVOO vs. OO (refined)			
						GLU	↓			
						HbA1c	↓			
						BMI, BW	↓			
						HDL-C	↓			
						AST, ALT, Visfatin	↓			
						Fasting IL-6, TNF-α, hsCRP, Adiponectin, Apelin	↔			
**10**	[54]	2015	Hypercholesterolemic participants	25 mL/day of EVOO with a low phenolic content (80 ppm) vs. HP-EVOO (500 ppm) which was enriched with its own polyphenol and complemented with thyme phenolics using a phenol extract obtained from a mixture of freeze-dried olive cake and dried thyme. HP-EVOO contained 50% of olive polyphenol and 50% of thyme phenolics	3 weeks, randomized, double-blind, crossover, controlled trial, 33 hypercholesterolemic volunteers randomized, 2-week washout periods with a common OO	Biomarker	HP-EVOO vs. EVOO			
						HDL-C	↑			
						LCAT Activity	↑			
						CETP activity	↓			

HP-EVOO: High Polyphenol Extra Virgin Olive Oil, GSH: Reduced Glutathione, GSSG: Oxidized Glutathione, BW: body weight, HFD: high-fat diet, LFD: low-fat diet, MDA: Malondialdehyde, NAFLD: Nonalcoholic Fatty Liver Disease, AST: aspartate aminotransferase, ROS: Reactive Oxygen Species, T2DM: Type 2 Diabetes Mellitus or T2D: type 2 diabetes, TNF-α: tumor-necrosis factor-alpha, LDL-C: low-density lipoprotein-cholesterol, GLUT2: glucose transporter 2, TGs: triglycerides, GLU: fasting glucose, HDL-C: high-density lipoprotein-cholesterol, IL-10: interleukin 10, IL-6: interleukin 6, CXCL1: chemokine (C-X-C motif) ligand 1, CRP: C-Reactive Protein, IFN-γ: Interferon-γ, COX-2: cyclooxygenase-2, SFAs: saturated fatty acids, T-CHOL: total cholesterol, UA: Uric Acid, Cr: Creatinine, OO: olive oil, FFA: free fatty acid, AKT: protein kinase B, IL-1β: interleukin 1β, VCAM-1: vascular cell adhesion molecule 1, ADP: Adenosine Diphosphate, TRAP: Thrombin Receptor Activating Peptide, BMI: body mass index, HbA1c: hemoglobin A1C, IL-12p40: interleukin 12p40, IL-1RA: interleukin 1 receptor antagonist, WC: waist circumference, DBP: Diastolic Blood Pressure, SBP: systolic blood pressure, LPS: Lipopolysaccharide, ALT: Alanine Transaminase, MetS: metabolic syndrome, ET-1: Endothelin-1, ACE: Angiotensin-converting enzyme, NR1H2: Nuclear Receptor Subfamily 1 Group H Member 2, IL8RA: interleukin 8 receptor antagonist, hs-CRP: High-Sensitivity C-Reactive Protein, LCAT: Lecithin-Cholesterol Acyl-Transferase, PON: Paraoxonase, CETP: cholesterylester transfer protein, FC: Free Cholesterol, EC: Esterified Cholesterol, PLs: Phospholipids. In table, the symbol (↑) indicates an increase, the symbol (↓) indicates a decrease and the symbol (↔) indicates no differences.

## 4. Conclusions

This scoping review highlights the beneficial effects of high phenolic extra virgin olive oil (HP-EVOO) on various biological functions, while a great number of studies indicate the superiority of HP-EVOO cardiometabolic effects compared to lower phenolic olive oils. Evidence from humans and animal models has shown that the consumption of HP-EVOO improves lipidemic profile and glycemic control and reduces systematic inflammation and oxidative stress. Therefore, HP-EVOO consumption may offer enhanced protection against metabolic syndrome, hypertension, type 2 diabetes and NAFLD. HP-EVOO seems to regulate expression of genes implicated in inflammation and metabolism of lipids and glucose. Moreover, the antiplatelet effect of HP-EVOO and the improvement in HDL functionality underline its strong contribution to the reduction in cardiovascular risk. HP-EVOO is characterized by a more pronounced benefit in enhancing endothelial function and antioxidant defense—two important aspects that contribute to public health. Follow-up research will further examine the possible role of HP-EVOO polyphenols in modulating the formation and biological actions of advanced glycation end products (AG-Es), which are recognized to be significant contributors to metabolic and vascular complications of obesity and type 2 diabetes.

Overall, the evidence here shows that HP-EVOOs represent an essential strategy for the prevention and treatment of cardiometabolic diseases and, in general, cardiovascular risk.

## Data Availability

All data and analysis are available within the manuscript and/or upon request to the corresponding author.

## Supplementary Materials

The following supporting information can be downloaded at: https://www.mdpi.com/article/10.3390/clinpract15030054/s1, Table S1: Preclinical data on the effects of high polyphenol extra virgin olive oil.
