# Supplementary material for: High Polyphenol Extra Virgin Olive Oil and Metabolically Unhealthy Obesity: A Scoping Review of Preclinical Data and Clinical Trials
Source: Clin Pract. 2025 Mar 7;15(3):54. doi: 10.3390/clinpract15030054 (PMC11941193; doi:10.3390/clinpract15030054)
Supplement: Supplementary file 1 [file clinpract-15-00054-s001.zip › clinpract-3491672-supplementary.pdf]

High Polyphenol Extra Virgin Olive Oil and Metabolically Unhealthy Obesity: A Scoping Review of Preclinical Data and Clinical Trials

Supplementary Material

Supplementary Table 1: Preclinical data on the effects of high polyphenol extra virgin olive oil.

a/a	Ref.	Year	Animal Model	Dosage	Design	Effect		
**1**	[36]	2022	C57BL/6N, weight-matched male mice	HP-EVOO consumption in positive group to be equal to 0.10 ± 0.01 g/mouse/day	19 mice fed *ad libitum* for 16 weeks: 1) SFA diet; n = 9) or 2) HP-EVOO diet; n = 10)	Biomarker	HP-EVOO *vs.* SFA	
						BW gain	↓ (albeit non-significant)	
						Urine and plasma polyphenols	↑	
						Plasma GSH	↔	
						Plasma GSSG	↓	
						Circulating proline	↓	
**2a**	[41]	2021	Wistar rats rendered diabetic by single dose of alloxan monohydrate (65 mg/kg body weight	10 mL/kg BW of HP-EVOO (442.321 ± 6.73 mg/kg total phenols) daily for 30 days	6 healthy rats were treated with distilled water (10 ml/kg BW), 6 healthy rats were treated with HP-EVOO (10 ml/kg BW)	Biomarker	HP-EVOO *vs.* Distilled water	HP-EVOO *vs.* Thyme Honey
						Blood GLU	↔	↔
						BW	↔	↔
						Hepatic Enzymes levels	↔	↔
						T-CHOL, LDL-C, TG	↔	↔
						Urea, UA, Cr	↔	↔
						Enzymatic antioxidants	↔	↔
						MDA	↔	↔
						Histopathological tissue changes of liver, kidney, pancreas	↔	↔
**2b**	[41]	2021	Wistar rats rendered diabetic by single dose of	10 mL/kg BW of HP-EVOO (442.321 ± 6.73 mg/kg total	6 diabetic rats were treated with distilled water (10 ml/kg BW), 6	Biomarker	HP-EVOO *vs.* Distilled water	HP-EVOO *vs.* Thyme Honey
						Blood GLU	↓	↓
						BW	↔	↔
						Hepatic Enzymes	↓	↓

			alloxan monohydrate (65 mg/kg body weight	phenols) daily for 30 days	diabetic rats were treated with HP-EVOO (10 ml/kg BW)	T-CHOL, LDL-C, TG	↓	↓
						Urea, UA, Cr	↓	↓
						Enzymatic Antioxidants	↑	↓
						GSH	↑	↓
						GPx	↑	↓
						MDA	↓	↓
						Histopathological tissue changes of liver, kidney, pancreas	↓	↓
**3**	[37]	2021	Female *Ldlr*−/−.Leiden mice	HFD with HP-EVOO group: 6.08 mg of polyphenols/kg of mouse BW/day	68 mice with food and water *ad libitum* for 32 weeks were randomly divided into four groups with a) LFD b) HFD and Lard, c) HFD with EVOO and d) HFD with HP-EVOO	Biomarker	HFD-HP-EVOO *vs.* HFD-Lard	HFD-HP-EVOO *vs.* HFD-EVOO
						BW gain	↓	↑
						Blood GLU Metabolism	↑	↓
						Insulin Sensitivity	↑	↑
						T-CHOL, HDL-C	↑	↑
						LDL-C	↑	↔
						Liver steatosis	↓	↔
						Liver fibrosis	↓	↑
**4**	[38]	2019	Five-week-old C57BL6J male mice	EVOO: 104 mg polyphenols/L and HP-EVOO: 447 mg polyphenols/L	120 mice were fed food and water *ad libitum* for 36 weeks. For the first 12 weeks, they were divided into two groups: a control LFD group (n =	Biomarker	HFD-HP-EVOO *vs.* HFD-Lard	HFD-HP-EVOO *vs.* HFD-EVOO
						BW	↓	↔
						Fasting glycemia, insulinemia and insulin resistance	↓	↔
						Insulin sensitivity	↑	↔
						β-cell apoptosis	↓	↔
						β-cell number	↑	↔

					30) and a Lard-based diet group (n = 90). After 12 weeks, the LFD group continued on chow for another 24 weeks, while the HFD group was divided into three groups (Lard, HP-EVOO, OO) for 24 more weeks.			
**5**	[42]	2018	HLA-B27 transgenic rats and F344 Wild-type rats	HP-EVOO: 4.3 mg of polyphenols /kg of rat BW/day EVOO: 0,056 mg of polyphenols /kg of rat BW/day	20 rats were fed *ad libitum* diet containing 10% Corn oil group, n=6 or HP-EVOO group, n=7 or EVOO group, n=7 for 3 months	Biomarker	HP-EVOO *vs*. EVOO	
						TNF-α gene expression	↓	
						T-CHOL	↓	
						TG	↔	
						Inflammatory responses	↔	
**6**	[39]	2018	Female *Ldlr*−/−.Leiden mice	EVOO group: 79 mg/kg total phenolic compounds. HP-HP-EVOO group: 444 mg/kg total	74 mice were randomly divided into four groups. One group was fed a standard LFD; *n* = 20, 13% of total	Biomarker	HFD-HP-EVOO *vs*. HFD-Lard	HDF-HP-EVOO *vs*. HFD-EVOO
						Adipocyte hypertrophy	↓	↔
						Adipose tissue inflammation	↓	↑
						Production of peroxynitrites and inflammatory markers	↓	↔

				phenolic compounds	kcal came from fat and three groups were fed HFD (48% of the total kcal came from fat): HFD based on Lard, n = 21) HP-EVOO, n = 14, or EVOO, n = 19	Adiponectin	↑	↓
						Atherosclerotic lessions severity	↓	↑
**7**	[43]	2016	Male C57 BL/6 J mice	100 mg/d HP-EVOO with different polyphenolic concertation: EVOO I: EVOO containing 116 mg total polyphenols/kg oil. (HP-EVOO II: EVOO containing 407 mg total polyphenols/kg oil. HP-EVOO III: EVOO containing 859 mg total polyphenols/kg oil	Weaning mice were randomly assigned to one of four groups, supplemented with HP-EVOO with increasing polyphenols concentration. Control Diet (CD) Groups: Control Diet: Baseline control with no EVOO supplementation, EVOO I, HP-EVOO II HP-EVOO III	Biomarker	HP-EVOO *vs.* Control Diet and EVOO I, EVOO II	HFD-HP-EVOO *vs.* HFD and HFD-EVOO I, HFD-HP-EVOO II
						Enzymatic Antioxidants	↔	↑
						Adipose tissue ω-3, TG, PUFA	↔	↓
						ω-6/ω-3 ratio	↔	↓
						Hepatic fat accumulation, TG, FFA	↔	↓
						Lipogenic enzymes	↔	↓
						Lipolytic enzymes	↔	↑
						HDL-C	↔	↑
						HDL/LDL ratio	↔	↑
						Serum levels of fasting insulin	↔	↓

**8**	[40]	2016	Male C57BL/6J mice	EVOO: 104 mg polyphenols/L and HP-EVOO: 447 mg polyphenols/L	Mice (three animals from each group were analyzed and each run was repeated at least twice). (5-week-old) fed a standard diet or a Lard-based diet for 12 weeks to develop NAFLD. High fat fed mice were then divided into four groups and fed for 24 weeks with: Lard; HP-EVOO; EVOO	Biomarker	HFD-HP-EVOO *vs.* HFD-Lard	HFD-HP-EVOO *vs.* HFD-EVOO
						BW gain	↓	↓
						MUFAs (especially oleic acid)	↑	↑
						SFAs	↓	↓
						T-CHOL, LDL-C	↓	↓
						HDL-C	↑	↑
						Plasma and epididymal fat INF-γ, IL-6, Leptin	↓	↓
						Hepatic lipid accumulation and fibrosis	↓	↓
						NAFLD activity scores	↓	↓
**9**	[45]	2016	Male Sprague-Dawley rats	0,290 mg of polyphenols /kg of rat BW/day	24 Male Sprague-Dawley rats were divided into four groups (n = 6 for each group) based on the different types of diet, as following: standard diet, HFD, HP-EVOO	Biomarker	HP-EVOO *vs.* HFD	HP-EVOO *vs.* OO without polyphenols
						Increased levels of serum TG and ALT and total liver lipids induced by HFD	↓	↓
						Phosphorylation of AKT and GLUT2 expression in liver	↑	↑
						mRNA levels of TNF-α, COX-2	↓	↓

					diet and OO without polyphenols diet	MDA	↓	↓			
						Intronization of proteins, ROS production	↓	↓			
						Serum levels of adiponectin, leptin	↓	↓			
						Proinflammatory (TNF-α and IL-1β)	↓	↓			
						Anti-inflammatory mediators (IL-10)	↑	↑			
**10**	[46]	2016	8-week-old albino Wistar rats	Total phenolics per each oil and oil product: EVOO: 168 mg/Kg, Sunflower Oil enriched with EVOO: 302 mg/Kg, High-Oleic Sunflower Oil: normal, High-Oleic Sunflower Oil enriched with EVOO: 341 mg/Kg.	64 male 8-week-old albino Wistar rats were randomly divided into eight groups (A–H) of eight rats each and fed different diets for nine weeks: Group A (control) received laboratory chow pellets; Group B received laboratory chow pellets supplemented with 2% cholesterol (high-cholesterol diet).	Biomarker	HP-EVOO *vs.* High Cholesterol Diet	HP-EVOO *vs.* EVOO phenolic deprived	HP-EVOO *vs.* Sunflower Oil and High-Oleic Sunflower Oil	HP-EVOO *vs.* Sunflower Oil and High-Oleic Sunflower Oil enriched with EVOO	
						Serum TC, LDL-C	↑	↑	↑	↑	
						Serum TG	↓	↑	↑	↑	
						Heart TNF-α, MDA	↓	↓	↓	↓	
						Heart a-tocopherol	↑	↔	↓	↓	
**11**	[49]	2015				Biomarker	HP-EVOO *vs.* High Cholesterol Diet	HP-EVOO *vs.* EVOO phenolic deprived	HP-EVOO *vs.* Sunflower Oil and High-Oleic	HP-EVOO *vs.* Sunflower Oil enriched	HP-EVOO *vs.* High-Oleic Sunflower Oil enriched

					The other six groups (C–H) were fed high-cholesterol diet supplemented with 10% oils or oil products: Group C received EVOO; Group D received phenolics-deprived EVOO; Group E received sunflower oil; Group F received sunflower oil enriched with EVOO; Group G received high-oleic sunflower oil; Group H received high-oleic sunflower oil enriched with EVOO				Sunflower Oil	with EVOO	with EVOO
						E-selectin	↓	↔	↓	↓	↔
						VCAM-1	↓	↔	↓	↓	↓
						MDA	↓	↓	↓	↓	↔
						T-CHOL, LDL-C	↑	↔	↑	↑	↑
						TG	↔	↑	↑	↑	↑
						HDL-C	↓	↔	↔	↔	↔

HP-EVOO: High Polyphenol Extra Virgin Olive Oil, GSH: Reduced Glutathione, GSSG: Oxidized Glutathione, BW: Body Weight, HFD: High Fat Diet, LFD: Low Fat Diet, MDA: Malondialdehyde, NAFLD: Nonalcoholic Fatty Liver Disease, AST: Aspartate Aminotransferase, ROS: Reactive Oxygen Species, T2DM: Type 2 Diabetes Mellitus or T2D: Type 2 Diabetes, TNF-α: Tumor-Necrosis Factor-alpha, LDL-C: Low-Density Lipoprotein-Cholesterol, GLUT2: Glucose Transporter 2, TG: Triglycerides, GLU: Fasting glucose, HDL-C: High Density Lipoprotein-Cholesterol, IL-10: Interleukin 10, IL-6: Interleukin 6, CXCL1: chemokine (C-X-C motif) ligand 1, CRP: C-Reactive Protein, IFN-γ : Interferon-γ,

COX-2: Cyclooxygenase-2, SFA: Saturated Fatty Acids, T-CHOL: Total cholesterol, UA: Uric Acid, Cr: Creatinine, OO: Olive Oil, FFA: Free Fatty Acid, AKT: Protein Kinase B, IL-1β: Interleukin 1β, VCAM-1: Vascular Cell Adhesion Molecule 1, ADP: Adenosine Diphosphate, TRAP: Thrombin Receptor Activating Peptide, BMI: Body Mass Index, HbA1c: Hemoglobin A1C, IL-12p40: Interleukin 12p40, IL-1RA: Interleukin 1 receptor antagonist, WC: Waist Circumference, DBP: Diastolic Blood Pressure, SBP: Systolic Blood Pressure, LPS: Lipopolysaccharide, ALT: Alanine Transaminase, MetS: Metabolic Syndrome, ET-1: Endothelin-1, ACE: Angiotensin-converting enzyme, NR1H2: Nuclear Receptor Subfamily 1 Group H Member 2, IL8RA: Interleukin 8 receptor antagonist, hs-CRP: High-Sensitivity C-Reactive Protein, LCAT: Lecithin-Cholesterol Acyl-Transferase, PON: Paraoxonase ,CETP: cholesterylester transfer protein, FC: Free Cholesterol, EC: Esterified Cholesterol, PL: Phospholipids. In table, the symbol (↑) indicates an increase, the symbol (↓) indicates a decrease and the symbol (↔) indicates no differences.
